# Quantifying endothelial cell proliferation in the zebrafish embryo

**DOI:** 10.12688/f1000research.73130.1

**Published:** 2021-10-11

**Authors:** George Bowley, Timothy JA Chico, Jovana Serbanovic-Canic, Paul C Evans

**Affiliations:** 1 Department of Infection, Immunity and Cardiovascular Disease, University of Sheffield, Sheffield, UK; 2 Bateson Centre, University of Sheffield, Sheffield, UK

**Keywords:** Zebrafish, Endothelial Cell, Proliferation, Microscopy

## Abstract

**Introduction**: Endothelial cell (EC) proliferation is a fundamental determinant of vascular development and homeostasis, and contributes to cardiovascular disease by increasing vascular permeability to blood-borne lipoproteins. Rodents have been traditionally used to analyse EC proliferation mechanisms in vascular health and disease; however, alternative models such as the zebrafish embryo allow researchers to conduct small scale screening studies in a physiologically relevant vasculature whilst reducing the use of mammals in biomedical research. *In vitro* models of EC proliferation are valuable but do not fully recapitulate the complexity of the *in vivo* situation. Several groups have used zebrafish embryos for vascular biology research because they offer the advantages of an *in vivo* model in terms of complexity but are also genetically manipulable and optically transparent.

**Methods: **Here we investigated whether zebrafish embryos can provide a suitable model for the study of EC proliferation. We explored the use of antibody, DNA labelling, and time-lapse imaging approaches.

**Results: **Antibody and DNA labelling approaches were of limited use in zebrafish due to the low rate of EC proliferation combined with the relatively narrow window of time in which they can label proliferating nuclei. By contrast, time-lapse imaging of fluorescent proteins localised to endothelial nuclei was a sensitive method to quantify EC proliferation in zebrafish embryos.

**Discussion: **We conclude that time-lapse imaging is suitable for analysis of endothelial cell proliferation in zebrafish, and that this method is capable of capturing more instances of EC proliferation than immunostaining or cell labelling alternatives. This approach is relevant to anyone studying endothelial cell proliferation for screening genes or small molecules involved in EC proliferation. It offers greater biological relevance than existing *in vitro *models such as HUVECs culture, whilst reducing the overall number of animals used for this type of research.

Research highlights **Scientific benefit(s)**

• Endothelial cell (EC) proliferation can be visualised at the single cell level in live embryos.

**3Rs benefit(s)**

• Zebrafish embryos can be used to partially replace mouse models in early cardiovascular disease research.
• This time-lapse zebrafish embryo proliferation assay is a non-terminal refinement ofimmunolabelling and cell labelling approaches, which require tissue fixation.

**Practical benefit(s)**

• Only one tank of adult zebrafish are required to produce transgenic embryos for this method (20-40).
• Fewer than 30 embryos are required for time-lapse experiments, thus fewer adult pairs are required which reduces breeding stress.
• Use of transgenic zebrafish lines allows for proliferating cells to be visualised in real-time without use of embryo fixation, compared to immunolabelling and cell labelling approaches which require embryo fixation.
• The zebrafish EC proliferation assay allows simultaneous quantification of proliferation in up to 24 embryos. Assuming four embryos are used as controls, this makes it possible to compare four treatment groups where each group has n = 5 animals. A comparable experiment using 24 mice would require each mouse to be culled, which incurs significant additional cost and effort, and may be difficult to justify, particularly for screening experiments where the potential benefits of the research are unknown.

**Current applications**

• Screening for genes involved in EC proliferation in response to blood flow.

**Potential applications**

• As a screening assay to identify new drugs/agents that prevent pro-atherogenic changes in endothelial cell proliferation in response to flow.
• Screening for genes involved in migration and apoptosis in response to flow by observing cell movement throughout the time-lapse, effects on apoptosis could be screened by counting the number of ECs undergoing apoptosis either by looking for blebbing of EC nuclei, or using a different transgenic.

## Introduction

Endothelial cell (EC) proliferation is vital for vascular development and homeostasis, but is also associated with increased vascular permeability which is a key driver of atherosclerosis. ^1^ Cell culture and mouse models are widely used to study EC proliferation in atherosclerosis yet use of these models involves a trade-off between biological relevance, maintenance cost, and technical complexity depending on the model chosen. This paper explores the efficacy of immunostaining, molecular labelling, and transgenic approaches to study EC proliferation in zebrafish embryos.

Like most forms of human disease research, cardiovascular disease (CVD) studies conventionally identify genes for in-depth research by first applying wider screening approaches. The models used in these types of research have several advantages and disadvantages. *In vitro* models can be used for high throughput analysis of the genes and pathways that control EC proliferation, but do not model the interaction of different tissues and are therefore reductionist. Conversely, rodents and other mammals have provided physiologically relevant models, such as the ApoE mutant mouse line which is the *de facto* model of plaque formation in CVD research. Whilst mice are an excellent model for study of genes involved in atherosclerosis (indeed, based on the number of mouse vascular mechanics papers on PubMed, 30,000 mice were used for this research in the past decade), they are unsuitable for screening multiple genes at once as generating mutants is costly, time consuming and may be difficult to justify due to concerns about the effect of mutations on animal welfare and the number of animals that would be required in such a study. Zebrafish embryos survive by oxygen and nutrient diffusion, thus mutations which are embryo lethal in mammals such as abolished development of ECs ( *cloche*) or targeted knock-down of cardiac troponin ( *sih*) are not lethal in zebrafish embryos. These characteristics mean zebrafish embryos are uniquely amenable to study of genes involved in ECs, and EC responses to flow.

Whilst zebrafish embryos are not protected prior to 5 days post fertilization (dpf) in the UK, they are still live organisms and therefore present a partial replacement to use of other animals in CVD research. Nonetheless, the advantages of using zebrafish over *in vitro* models are multiple. One pair of adult zebrafish can produce hundreds of embryos in one day, and with just an incubator and E3 medium these embryos will each develop a complete cardiovascular system in two days, ^2^ with zero human intervention. These characteristics give studies on zebrafish embryos simplicity comparable with that of two-dimensional *in vitro* models, far greater simplicity than comparable three-dimensional *in vitro* models, and comparable biological relevance to mammalian *in vivo* models but with reduced cost and reduced detriment to animal welfare.

The zebrafish embryo is ideal for studying vascular biology, it is small, optically transparent, and allows manipulation of gene expression using morpholino oligonucleotides ^3^ (MOs), CRISPR-Cas9 ^4^ and CRISPR-Interference. ^5^ These characteristics allow rapid generation of genetically modified embryos and quantitative analysis of EC behaviour. With the exception of CRISPR-Cas9, these genetic approaches and quantitative methods are not viable for CVD research in mice. Whilst CRISPR-Cas9 is effective in mice, generation of a range of mutants for screening purposes remains infeasible for reasons described above.

Zebrafish have been used to model EC apoptosis and migration in response to blood flow. ^6^ ^–^ ^8^ Whilst these events can be reliably observed using existing methods, EC proliferation is usually estimated by counting the total number of ECs. However, the number of ECs is determined by a complex interaction of multiple factors, including apoptosis, cell migration and differentiation/dedifferentiation, and therefore cell number is not a reliable metric for studying proliferation. It is therefore important to identify a reliable and direct assay of EC proliferation in zebrafish. Here we describe our attempts to directly quantify EC proliferation in zebrafish embryos using a combination of techniques currently used for *in vivo* and *in vitro* models; immunohistochemistry (a technique widely used to study protein expression in mouse endothelium using the *en face* technique), DNA labelling (used in cell culture and mice) and time-lapse imaging, to determine which method detected the greatest number of proliferating ECs. Indirect approaches for studying proliferation such as cell number counting, viability, and metabolic activity assays were not pursued as their results can be influenced by migration and apoptosis.

## Methods

### Zebrafish maintenance and husbandry

Zebrafish care and experimental procedures were carried out under Project Licence 70/8588 issued by the UK Home Office and local ethical committee approval was obtained. Adult zebrafish were kept at a constant temperature of 28 ± 1°C and at pH 7.5 ± 0.5. They were subjected to a light/dark cycle of 14 hours of light and 10 hours of darkness. Adult zebrafish were fed a diet of artemia and dry Zebrafeed™. For mating, pairs of male and female zebrafish were placed into mating tanks and separated by a divider to allow accurate timing of fertilisation. For experiments where this was not required, a box for embryo collection with a mesh insert and marbles on top was placed in the fish tank to harvest progeny. The Nacre line ^9^ and transgenic (Tg) ( *fli1a:nls-mCherry*), ( *fli1a:LifeAct-mClover*), ^10^ and ( *fli1:EGFP*) lines were used. ^11^ ^,^ ^12^ Nacre zebrafish lack melanocytes and are thus more transparent than conventional wildtype lines, this makes Nacre highly suitable for light microscopy. Both transgenic lines use Nacre as background. Tg *fli1a:nls-mCherry* expresses a red fluorescent protein linked to a nuclear localisation sequence under the endothelial promoter, resulting in red fluorescence in EC nuclei. Tg *fli1a:LifeAct-mClover* expresses a f-actin:mClover fusion protein under the endothelial promoter, resulting in visualisation of the EC actin cytoskeleton. Initially, we intended to compare EC proliferation in embryos with and without blood flow, thus for early experiments (whole mount immunostaining) heart contraction was inhibited by silencing troponin T2A by injection of *tnnt2a* ATG morpholino at 3 ng final dose (sequence 5′-CATGTTTGCTCTGATCTGACACGCA-3′). As EC proliferation was not detected at this experimental stage, flow and no-flow comparison was stopped to minimise our use of embryos and only embryos with blood flow were used for subsequent experiments.

### Zebrafish welfare and use

Adults: For all experiments described, embryos were produced by pair mating eight male and eight female adults of the same transgenic line. This was done to minimize the chance that zero pairs produced embryos, and to minimise confounders by limiting the genetic diversity of the embryos. Adults do not suffer pain or distress from pair mating and were returned to their home tanks immediately after producing embryos, home tanks contain environmental enrichments such as fake jellyfish and fake seaweed. No adults suffered adverse events as a consequence of this work.

Embryos: No embryos used in this study exceeded 5.2 dpf. For all experiments described, embryo collection stopped after 60 embryos were obtained. Sex of embryos was not determined (as is standard practice in zebrafish research). For PCNA and EdU experiments, embryos were treated then screened for the endothelial marker ( *Tg fli1:EGFP* for PCNA, and *Tg fli1a*: *nls-mCherry* for EdU) then three embryos were randomly selected for imaging. For time-lapse imaging experiments, embryos were screened for *Tg flia:nls-mCherry* and *Tg fli1a:LifeAct-mClover*, then three embryos were randomly selected for imaging. Embryos not selected for imaging were humanely destroyed using bleach.

### Whole mount immunostaining of proliferating cell nuclear antigen (PCNA)

Tg *(fli1:EGFP)* embryos were fixed in 4% paraformaldehyde (PFA) at 30 hpf to allow screening for proliferation in the main trunk vascular beds, then whole mount immunostaining was performed using anti-PCNA antibody (GeneTex, RRID: AB_11161916. Diluted 1:50) and AlexaFluor-568 mouse anti rabbit secondary antibody (ThermoFisher, RRID: AB_143165. Diluted 1:200). Colocalization of PCNA and *fli1*-EGFP was used to define proliferating ECs. Embryos not treated with PCNA antibody were imaged during protocol optimization to ensure the specificity of PCNA antibody. Detection of cell proliferation was defined as where a nucleus is both positive for PCNA and for *fli1*-EGFP.

### 5-Ethynyl-2′-deoxyuridine (EdU) labelling

The Click-iT™ EdU Cell Proliferation Kit (Thermo Fisher) was used to label DNA of proliferating cells with AlexaFluor-488. The protocol was modified for zebrafish as follows:

Zebrafish larvae with Tg ( *fli1a:nls-mCherry*) were collected at 30 hpf and incubated in EdU solution (EdU 500 uM, 15% DMSO, 85% E3) at 28°C for 1 h. Larvae were fixed in 4% PFA overnight then washed and transferred to permeabilization solution (supplied in kit) for 1 h. Permeabilised larvae were then treated according to manufacturer protocol. Proliferating ECs were defined as cells positive for *fli1a:nls*- *mCherry* and EdU.

### Brightfield microscopy

Standard light microscopy was used to screen out dead or undeveloped embryos, and to identify *tnnt2a* morphant embryos that lack a heartbeat. The morphant phenotype (no heartbeat) was observed in all (100%) of injected embryos, survival is indistinguishable from wildtype up to the time point tested (30 hpf).

### Fluorescent microscopy

Fluorescent microscopy was used to identify and select embryos containing *fli1a:nls-mCherry* or *fli1:EGFP* after they were non-terminally anaesthetised using E3 medium. Fluorescence screening was done using a ZEISS Axio Zoom V16 at 28 hpf. Zeiss filter set 63 (HE mRFP, cat no 489063-0000-000) was used. Camera mode was used and pixel binning set to 5 × 5 to maximise sensitivity to the fluorophore. Screened embryos were washed in E3 media without tricaine, then transferred into fresh E3 prior to further imaging.

### Confocal microscopy and time-lapse imaging

For confocal imaging, embryos were non-terminally anaesthetised using tricaine and mounted in 1–2% agarose over which standard E3 medium was placed. Still images were taken using a Nikon A1 confocal microscope at 1024 × 1024 resolution (0.62 μm per pixel), z-stacks were taken in 8-μm increments. Time lapses were taken using a Zeiss LSM880 in Airyscan mode. Images were captured at 1848 × 1848 resolution (0.47 μm per pixel), z-stacks were taken in 3.43-μm increments. Images were analysed using NIS Elements or ZEN respectively. Composite images were generated by performing maximum intensity projection of the stack data, no other processing was performed. For both microscopes, excitation was done by a 488 nm and 568 nm laser.

### Light sheet microscopy and time-lapse imaging

For light sheet microscopy, ^8^ ^,^ ^10^ non-terminally anaesthetised embryos were immobilised using 1% agarose and mounted in a glass capillary. Embryos were suspended in melted agarose (37°C, 0.8–1.2% w/v) then drawn up into a glass capillary using a plunger. Agarose suspended embryos were then pushed through the capillary and suspended into the imaging chamber containing E3 and tricaine. Images were captured at 1920 × 1920 resolution (0.23 μm per pixel), z-stacks were taken in 1-μm increments. Images were captured every 15 minutes for 4 h using ZEISS Z1 light sheet microscope and processed using ZEN software. Post-acquisition, maximum intensity projection was used to produce a single image for each time point. EC proliferation was defined as where *fli1a:nls-mCherry* nuclei visibly divide over the course of the time lapse. Experimental embryos were then euthanised using bleach.

### Statistical methods

The number of proliferation events detected using EdU labelling, time-lapse confocal microscopy and PCNA immunolabelling were compared using a two-way ANOVA (n = 3 embryos per each group). Each animal was treated as a single unit of analysis. Analysis was done using GraphPad PRISM (RRID:SCR_002798), an open-source alternative to this is JASP (RRID:SCR_015823).

## Protocols

### Materials

E3 medium: 5 mM NaCl, 0.17 mM KCL, 0.33 mM MgSO _4_ and 0.33 mM CaCl _2_ in dH _2_O. 4% PFA: paraformaldehyde diluted to 4% with E3 (One month shelf life when stored at 4°C). *tnnt2a* MO: diluted to a 100 μM stock with dH _2_O. Stock concentration should be determined using a Nanodrop and diluted to 3 ng/nL for microinjection. PBS: phosphate-buffered saline (ThermoFisher) diluted from 10× stock. PBST: phosphate-buffered saline with Tween-20. PBS made to 0.1% Tween-20. PDT: phosphate-buffered saline with 1% DMSO and 0.1% Triton X-100. TRIS buffer: tris base diluted in dH _2_O to 1 M stock. 150 mM solution was prepared from stock and pH adjusted to 8.0. Tricaine: 4 g tricaine methanesulfonate in 1 L dH _2_O pH 7–7.5. Blocking solution: 1% bovine serum albumin in PBS. Goat serum: goat serum stock (frozen) diluted to 10% in blocking solution. Bovine serum albumin (BSA): stock albumin powder should be added to dH _2_0 to make 10% (w/v) BSA solution, this solution should be aliquoted into 1.5-mL Eppendorf tubes and frozen for use as needed. Click-IT EdU AlexaFluor-488 Flow Cytometry Assay Kit – ThermoFisher C10420.

### Zebrafish lines

**Table:** 

Line name	Use case
***Tg fli1a:nls-mCherry***	Endothelial nuclear localised red fluorescence protein (RFP) marker.
***Tg fli1:EGFP***	Endothelial cytoplasmic green fluorescence protein (GFP) marker.
***Tg fli1a:LifeAct-mClover***	Endothelial f-actin GFP marker.
***Nacre***	Line in which described transgenes were inserted. Melanocytes are lacking, leading to improved optical clarity in embryos.

### Dechorionating zebrafish embryos

1) Collect embryos and transfer to a petri dish of E3 media.
2) Incubate embryos at 28°C until at least 24 hpf.
3) Insert forceps to gently pierce the embryo chorion (Sup. 1 – 1).
4) Gently open forceps to tear the chorion (Sup. 1 – 2).
5) Open forceps until embryo is free of the chorion (Sup. 1 – 3).

### Whole mount immunostaining for proliferating cell nuclear antigen (PCNA)

1) Collect *fli1-EGFP* embryos and transfer to a petri dish of E3 media then incubate at 28°C to 30 hpf.
2) Dechorionate embryos.
3) Fix in 4% PFA overnight at 4°C – now protect samples from light as far as possible until imaging.
4) Wash in room temperature PBST (Phosphate buffered saline with 0.1% Tween-20) on a rocker for three 10-min washes.
5) Wash in room temperature TRIS buffer (150 mM pH 8.0) on a rocker for 5 min.
6) Equilibrate embryos in TRIS buffer at 70°C for 15 min.
7) Wash in room temperature PBST on a rocker for two 5-min washes.
8) Rinse with distilled water on ice.
9) Wash in chilled acetone (−20°C) for 15 min.
10) Rinse with distilled water for three 5-min washes.
11) Block with goat serum (10%), BSA (2%), and PBT (phosphate buffered saline with 0.1% Triton X-100) for 4 h at 4°C.
12) Incubate with primary antibody overnight at 4°C (Dilute PCNA 1:250 in blocking solution).
13) Wash in PBT for four 30-min washes.
14) Incubate with secondary antibody (AF-568 anti-rabbit diluted 1:500 in blocking solution).
15) Wash in room temperature PBST five times for 5 min.
16) Mount as described in ‘Mounting individual zebrafish embryos for confocal imaging’.
17) Image using a confocal microscope as described above.

### EdU labelling

1) Collect Tg *fli1a:nls-mCherry* embryos and incubate at 28°C until 30 hpf.
2) Dechorionate embryos.
3) Incubate embryos in labelling solution (500 uM EdU, 15% DMSO, E3) at 28°C for 1 h.
4) Fix in 4% PFA at 4°C overnight.
5) Wash with PBST for three 5-min washes, rock samples each time.
6) Permeabilise in 1× saponin solution (Saponin solution is part of kit. diluted in PDT – Phosphate buffered saline with 1% DMSO, 0.1% Triton X-100) for 1 h at room temperature while rocking samples.
7) Incubate with reaction cocktail (219 μL PBS, 5 μL copper protectant, 1.25 μL fluorescent azide, 25 μL buffer additive) for 1 h in the dark at room temperature whilst rocking.
8) Wash five times in PBST for 5 min whilst rocking.
9) Mount as described in ‘Mounting individual zebrafish embryos for confocal imaging’.
10) Image using a confocal microscope as described above.

### Mounting individual zebrafish embryos for confocal imaging

1) Take treated embryos and mount on glass slides in vectashield (RRID:AB_2336789). Embryos must be laid flat on their side. This occurs naturally for young embryos (~30 hpf). If older embryos are used, the yolk may prevent embryos from lying flat, in this case the embryo yolk and head can be cut off using a scalpel.

### Live imaging EC proliferation in zebrafish embryos

1) Collect Tg *fli1a:nls-mCherry* embryos and incubate at 28°C until 26 hpf.
2) Dechorionate embryos using forceps.
3A) For live imaging using LSM, suspend embryo(s) in melted low-melt agarose (37°C 0.8–1.2%), then use a plunger to draw the embryo(s) into a glass capillary. Wait 2 min for the agarose to set, then suspend the embryos (within the agarose column) into the LSM by gently pushing the plunger.
3B) For confocal imaging, add embryo(s) to melted low melt agarose (37°C 0.8–1.2%) then draw up embryo(s) and decant into an Ibidi μ-Dish 35mm (Ibidi cat no – 81156). Use thin-tip forceps to gently push the embryo into a side-mounted position, then wait for agarose to set.
4) Image using appropriate microscope ensuring 587 nm excitation is active. Adjust magnification to visualise as much of the trunk as possible whilst retaining nuclear resolution (for the LSM880 and AxioObserver SPIM, magnification should be set to 20×). Z-range should traverse the entire trunk vasculature and Z-interval should be set no lower than 8 μm.
5) For euthanasia, discard embryos into bleach. For revival, return embryos to E3.

### Microinjection of zebrafish embryo

1) Collect embryos and transfer to 28°C E3 media.
2) Take a glass slide and place it in a petri dish so it is unable to move further to one side.
3) With a Pasteur pipette, draw up embryos then, holding the glass slide petri dish slightly tilted, dispense embryos along the edge of the glass slide (Sup. 2).
4) Prepare a glass needle (World Precision Instruments, cat no TW100F-4) using a Sutter P1000 glass capillary puller. Suitable settings are heat 475, pull 60, vel 80, time 135, pressure 500. Refer to the user manual for capillary pulling optimization.
5) Add injection media containing 3 ng/nL of *tnnt2a* morpholino to a glass needle and calibrate the ejection volume using a graticule (Breckland Scientific, Precision Stage Micrometer – S1).
6) Using a manipulator (World Precision Instruments M3301) and PicoPump (H. Saur PV820), inject 1 nL of morpholino into each embryo until desired number of treated embryos are obtained (some extra embryos should be treated to account for failure to develop and death).

## Results

**Figure 1.**
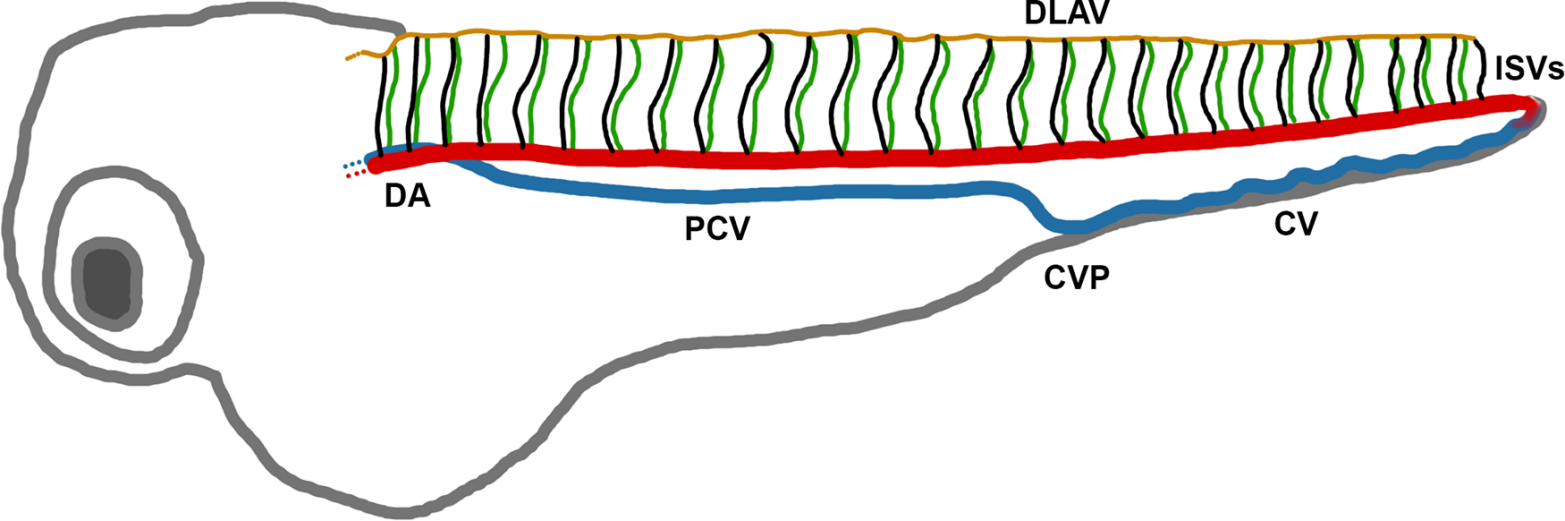
Diagram of the zebrafish vasculature at 72 hpf. DA – dorsal aorta. DLAV - dorsal longitudinal anastomotic vessel. CV - caudal vein. CVP - caudal vein plexus. PCV - posterior cardinal vein. ISVs - intersegmental vessels.

We analysed EC proliferation in the trunk vasculature of zebrafish embryos because this vascular bed allows simultaneous imaging of the intersegmental vessels and dorsal aorta ( Figure 1). We attempted to quantify EC proliferation in the zebrafish trunk using immunohistochemical, DNA labelling and transgenic approaches.

### Whole mount immunohistochemistry for PCNA was not effective for detecting EC proliferation

**Figure 2.**
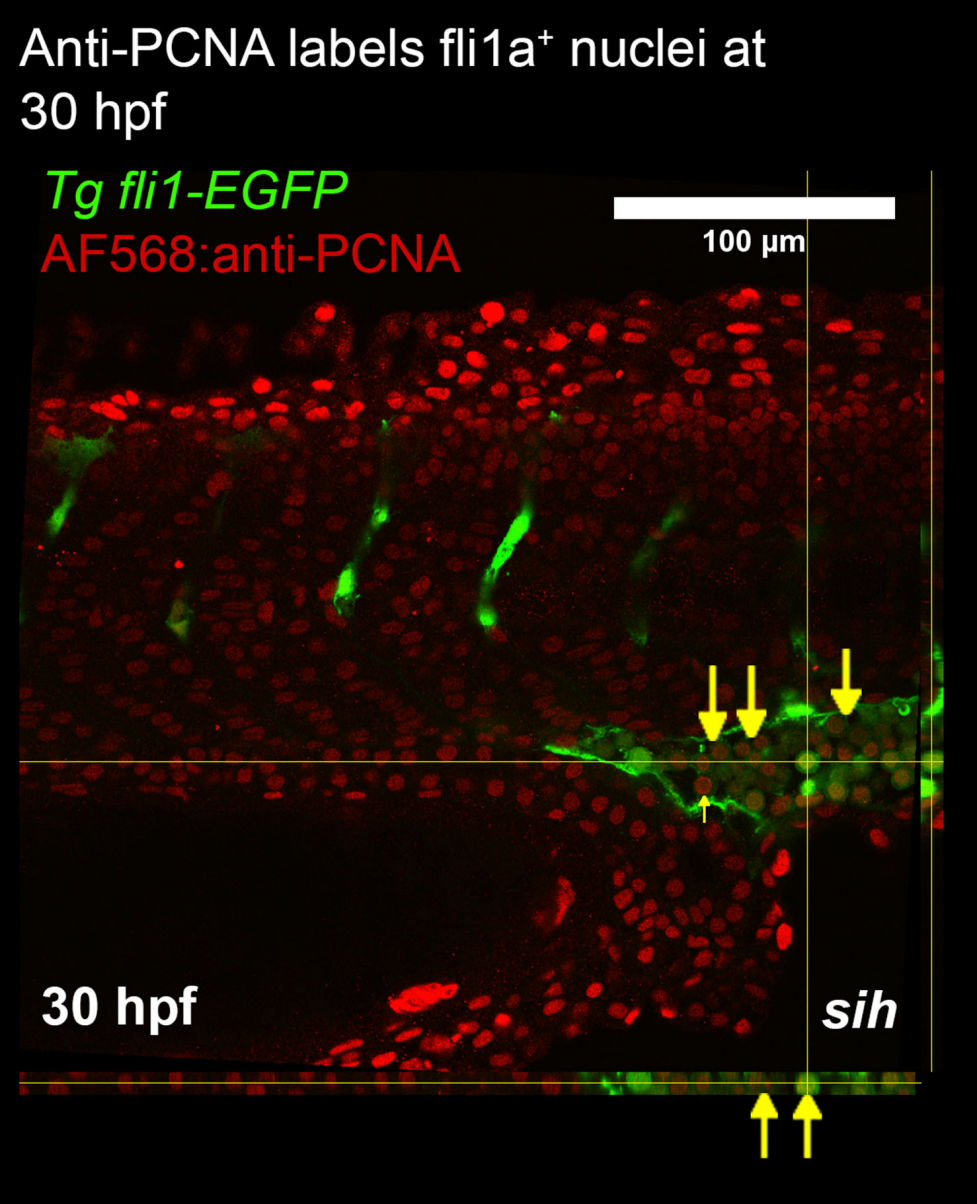
Whole mount immunostaining of zebrafish larvae for PCNA. Confocal imaging was used to generate a z-stack of fli1a-GFP and PCNA expression in the trunk. Orthogonal views are presented alongside the image. The caudal vein plexus and intersegmental vessels were imaged to determine whether endothelial cell proliferation was detected in these vascular beds. At 30 hpf anti-PCNA was colocalised with fli1a positive cells (labelled with yellow arrows). Since flow modifies EC proliferation, we studied embryos lacking blood flow (tnnt2a morphants; shown) and controls with normal blood flow. PCNA staining did not detect proliferating endothelial cells in either group. The pattern of staining suggests a deficiency of penetration of anti-PCNA antibodies into the centre of the embryo.

Immunohistochemistry can be used to label mitotic markers found in proliferating cells. We chose PCNA as a marker of proliferation for this study as an anti-PCNA antibody had been previously validated for immunostaining zebrafish kidney sections ^13^ and whole mount embryos. ^14^ To determine whether whole mount immunohistochemistry could detect EC proliferation, transgenic embryos expressing endothelial GFP ( *Tg fli1a:nls-EGFP*) were fixed at 30 hpf then treated with anti-PCNA antibody. Imaging of the embryo trunk detected many PCNA ^+^ cells in the caudal vein plexus (CVP) and none in the ISVs ( Figure 2).

### DNA labelling with EdU detected EC proliferation

**Figure 3.**
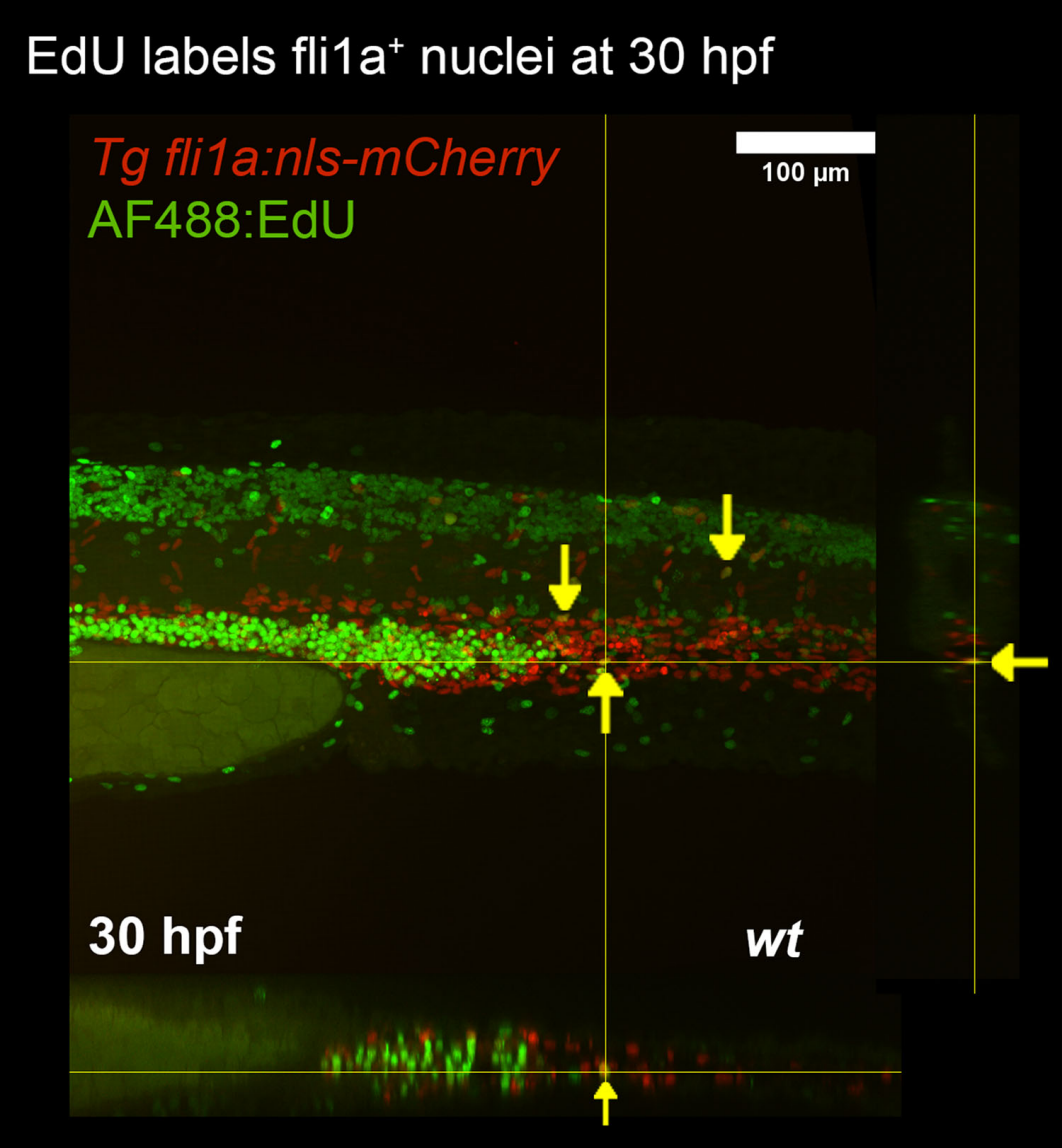
EdU labelling of zebrafish larvae. Proliferating ECs of Tg ( *fli1a:nls-mCherry*) embryos were labelled using EdU and visualised using EdU-binding fluorescent azide. Proliferating endothelial nuclei are marked (yellow arrows).Orthagonal views are presented for the X and Y axes.

The cell labelling approach works by incorporation of thymidine derivatives such as EdU into newly synthesised DNA. The modified thymidine is labelled with a fluorescent azide thereby enabling the identification of nuclei which have undergone proliferation. ^15^ An advantage of EdU labelling is that proliferation can be measured over a period of time ( *i.e.* the time that embryos are exposed to EdU), whereas immunostaining is restricted to detecting markers of proliferation at a single time point. To assess the effectiveness of EdU staining, transgenic embryos expressing endothelial nuclear localised mCherry (Tg *fli1a:nls-mCherry*) were treated with EdU from 30 to 31 hpf. Embryos were then fixed and treated with fluorescent azide. Between zero and three proliferating ECs were detected in the trunk of treated embryos, with detection occurring in the DA, but not the ISVs ( Figure 3).

### Time-lapse imaging of EC nuclei was a more sensitive assay of proliferation than PCNA or EdU staining

**Figure 4.**
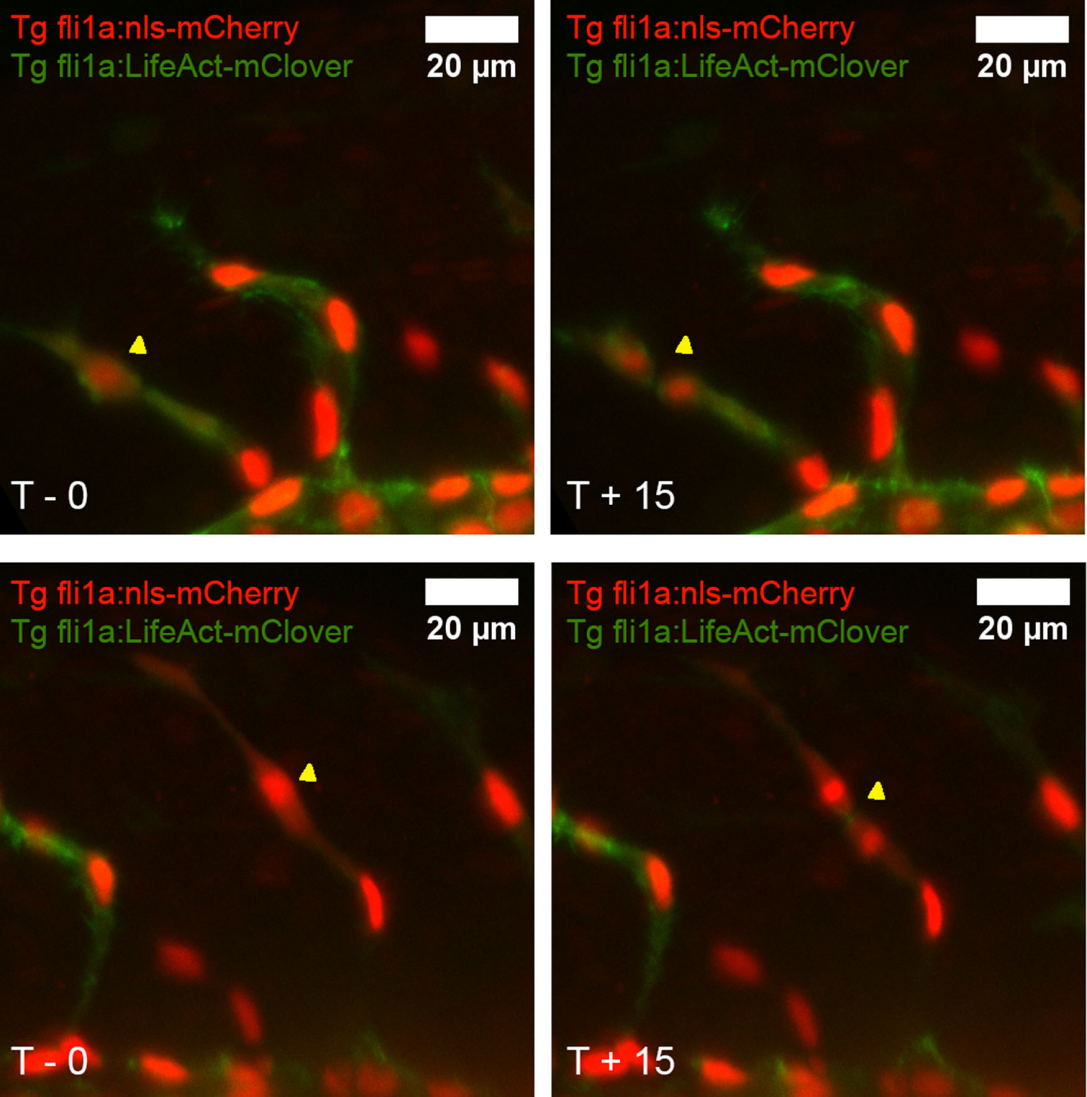
Endothelial cell proliferation can be directly observed in the intersegmental vessels. The trunk of WT zebrafish was studied from 26 to 30 hpf in 15-minute intervals using a light sheet microscope. Endothelial nuclei were labelled using the transgenic marker *fli1a:nls-mCherry* (red) whilst endothelial F-actin was labelled using *fli1a:lifeAct-mClover* (green). EC proliferation was defined as where one nucleus visibly divides into two (examples are shown and dividing cells are labelled with yellow arrows; compare upper left with upper right; compare lower left with lower right).

As previous approaches lacked specificity or very rarely detected EC proliferation,we sought a method for quantifying EC proliferation over a longer timeframe. EdU staining over prolonged time periods was not feasible because EdU can only reach vascular nuclei in the presence of permeabilization solvent dimethyl sulfoxide (DMSO) which has well described toxic effects above the 2% level. ^16^ As the 1-h EdU incubation protocol called for 15% DMSO, longer incubation times were not possible without reducing the DMSO concentration. We chose to pursue an alternative approach rather than further optimize EdU labelling. To do this, we carried out time-lapse imaging of transgenic embryos expressing endothelial nuclear localised mCherry ( *fli1a:nls-mCherry*) and cytoplasmic LifeAct-mClover ( *fli1a:LifeAct-mClover*) using a light sheet microscope. Using this approach, EC proliferation was quantified by counting the division of EC nuclei in all visible ISVs from 26 to 30 hpf ( Figure 4).

**Figure 5.**
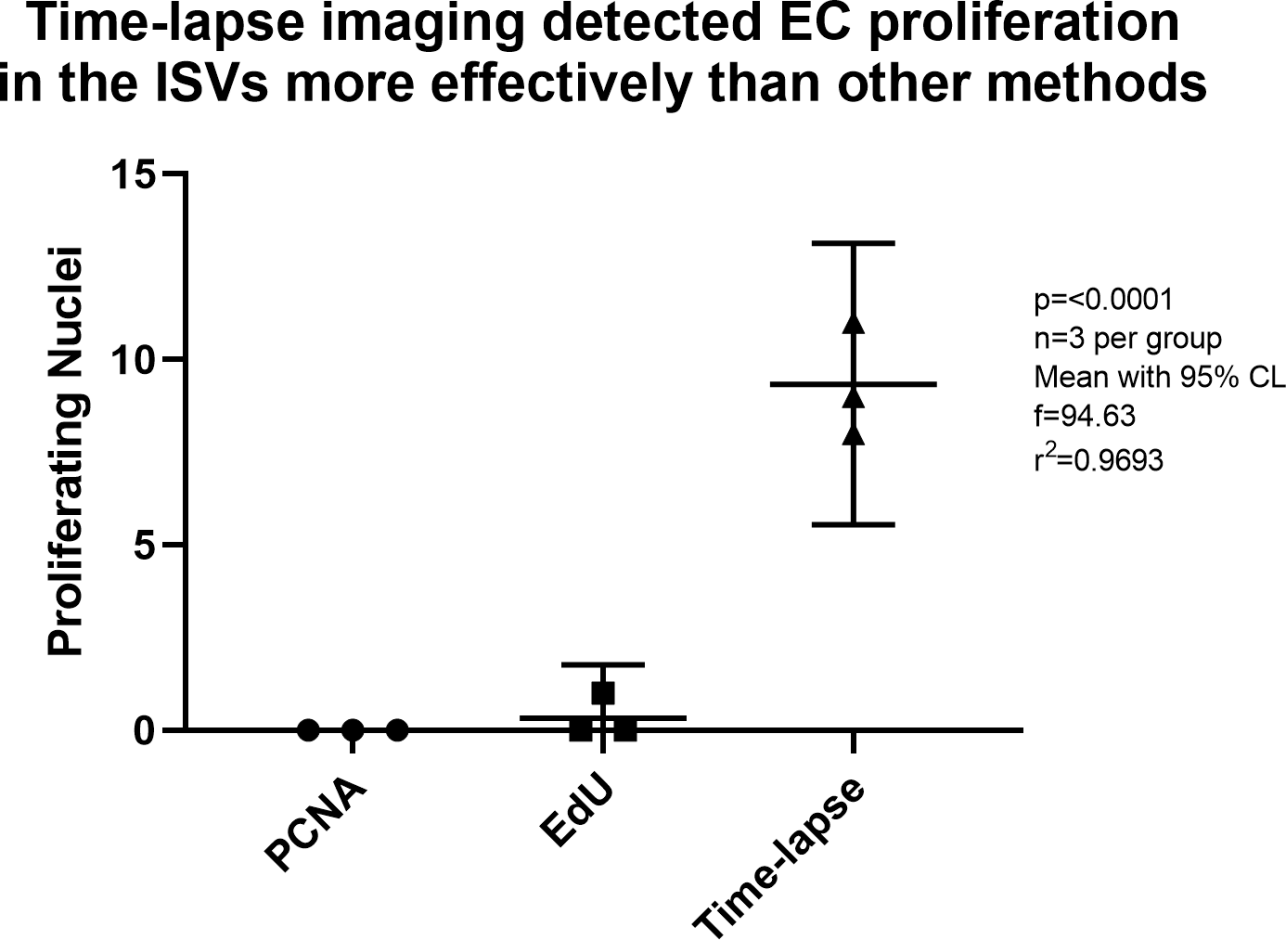
A comparison of detection of EC proliferation by PCNA immunolabelling, EdU cell labelling and time-lapse imaging of fluorescently labelled EC nuclei. Endothelial cell proliferation was studied at 30 hpf in PCNA treated embryos, 30 to 31 hpf in EdU treated embryos, and 29–45 hpf in time-lapse imaged embryos. Time-lapse imaging detected EC proliferation more effectively than other methods. Statistics were derived from analysis by ordinary one-way ANOVA.

Detection of EC proliferation in the intersegmental vessels was compared for each method. The ISVs are isolated from other tissues, so it was easiest to spot proliferation in this vascular bed. There are also relatively few nuclei in the ISVs, and ISV nuclei are less densely packed than nuclei in the DA/CVP. PCNA did not detect any proliferation events in the ISVs, EdU labelling detected fewer than one proliferation event on average, whereas time-lapse imaging detected an average of nine proliferation events ( Figure 5). Two-way ANOVA of the number of proliferation events detected by each method revealed a significant difference in detection across methods and no significant difference in detection across individual zebrafish embryos (p = 0.0002).

## Discussion

We tested three different methods for quantifying EC proliferation in the developing zebrafish embryos. Whole mount immunostaining for PCNA was effective for detecting proliferation *per se*, as evidenced by detection of PCNA positive nuclei ( Figure 2). A large number of PCNA positive nuclei were detected in the CVP at 30 hpf, however these cells were likely embryonic progenitors rather than ECs, which was confirmed in EdU labelling data where a large population of proliferating, non-endothelial cells were detected in the CVP. 30 hpf was chosen as at this point the embryo has a functional heart and has a functionally developed trunk vasculature. Study at later time points was avoided to minimize embryo size as others have shown that immunostaining for PCNA in zebrafish requires tissue sectioning, and adding this step would have made the protocol undesirably low throughput. Our findings are consistent with Luo *et al* ^14^ which suggested that use of antibodies to label deep tissues in whole mount zebrafish embryos may be difficult. This led us to seek a small-molecule approach for labelling proliferating ECs.

A DNA-labelling approach was tested in parallel. EdU is a small molecule that readily diffuses into cells in the presence of DMSO, during mitosis EdU is incorporated into newly synthesised DNA after which EdU can be detected by chemically bonding it to a fluorescent azide. As all components of the EdU labelling system were small molecules, we hypothesized that this technique would not be limited by penetration of reagents into deep tissues. Indeed, this was supported by our data as EdU labelling identified a population of proliferating cells in the caudal vein plexus that was not identified by the immunostaining approach ( Figure 3). Notably, whilst some EdU ^+^ ECs were identified, most EdU labelled cells were negative for the endothelial marker mCherry, and were likely haematopoietic progenitors which develop in the caudal vein plexus.

EdU labelling revealed that EC proliferation occurred at 0.25%–1% (1–3 nuclei per field of view containing 300–400 nuclei). This observation is comparable to those observed *in vitro* and mammalian systems which revealed EC proliferation at 0.67–0.75% of nuclei per hour ^17^ ^,^ ^18^; thus suggesting that EC proliferation rates are conserved between zebrafish embryos and mammalian systems.

**Figure 6.**
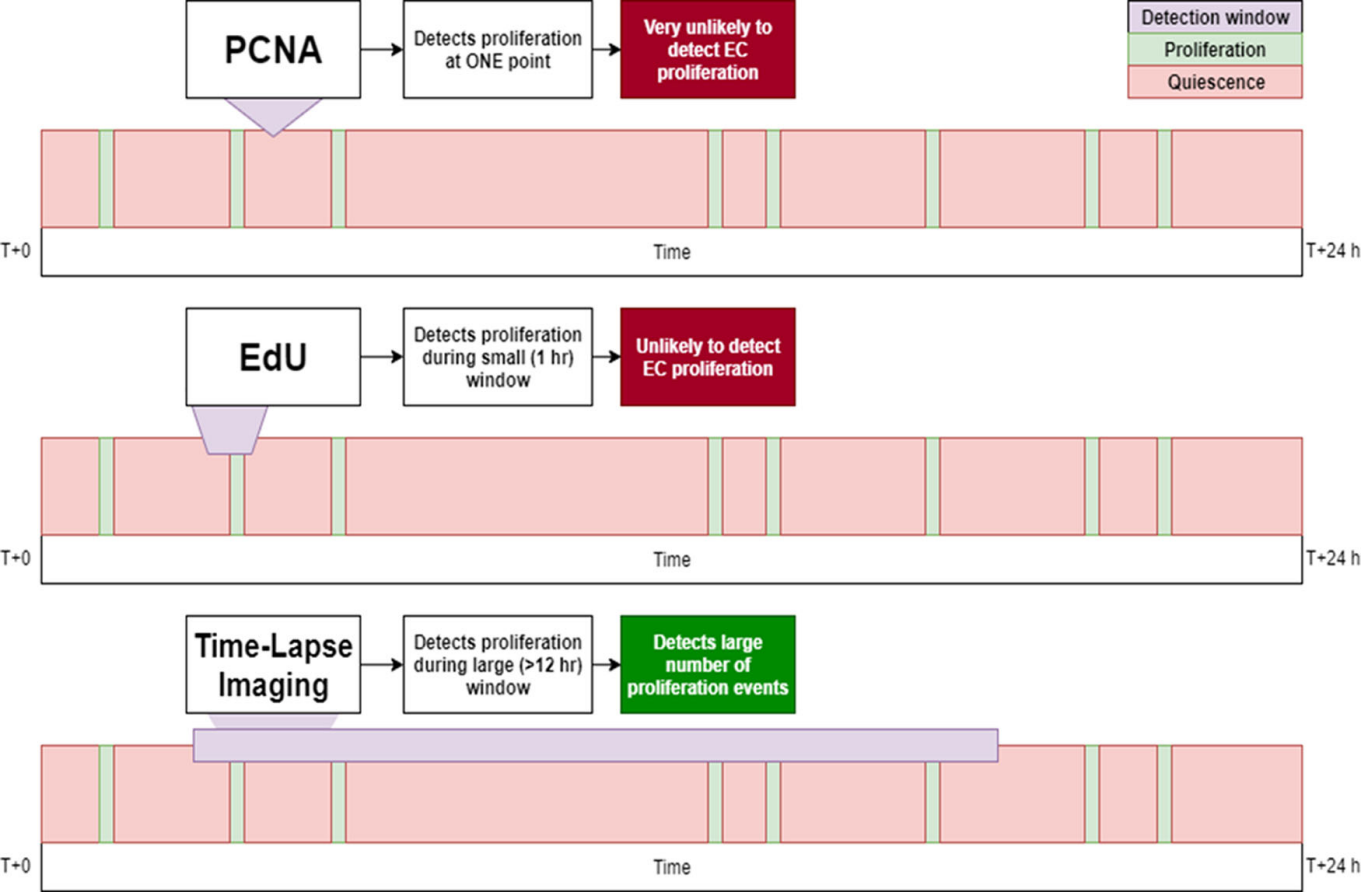
Diagram illustrating the effect of increasing the duration of analysis on the quantitation of endothelial cell proliferation. The PCNA approach uses a small (instantaneous) detection window, thus rarely detects proliferation events. EdU is more likely to detect proliferation but the window of detection remains too small to capture many events. Time-lapse imaging covers a much greater window of time and can therefore detect significantly more proliferation events.

**Figure 7.**
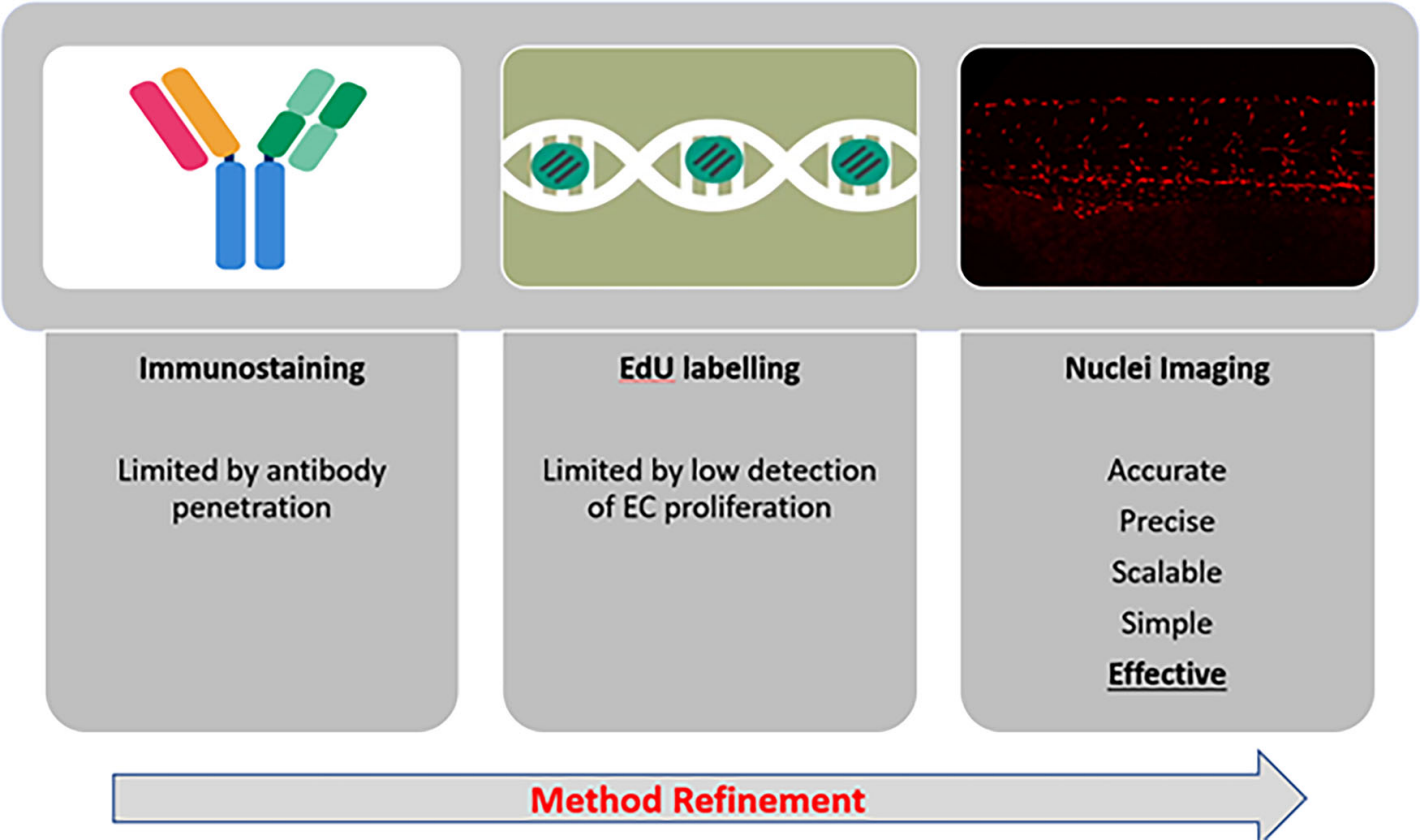
Graphical abstract. Immunostaining (for PCNA) is limited by antibody penetration. EdU labelling is limited by low detection of EC proliferation, the toxic effects of EdU treatment (which necessitates DMSO) prevent longer incubation times. Nuclei imaging by time-lapse microscopy is effective for studying EC proliferation.

Despite this, EdU labelling is unsuitable to analyse the effects of interventions on proliferating ECs because of the limited window of detection and low number of proliferating cells detected. Therefore, we sought a method to capture greater numbers of proliferating EC from zebrafish embryos. We found that light sheet time-lapse microscopy of transgenic embryos (Tg *fli1a:nls-mCherry, fli1a:lifeAct-mClover*) enabled direct visualisation of EC nuclei division in proliferation ( Figure 4). As this transgenic line expressed fluorescent protein exclusively in ECs it was relatively trivial to count the total number of EC proliferation events. This approach has several major advantages:

• Lower material costs, which eliminates the need for antibody or cell labelling products used in immunostaining or cell labelling approaches.
• Shorter experiment runtime, as experiments can be done as soon as embryos reach the desired stage of development compared to other approaches which require an additional period of time for fixation and treatment.
• Non-lethal procedure, embryos do not need to be fixed to conduct the experiment.
• Greater detection of EC proliferation compared to other zebrafish approaches (IHC, cell labelling) because of a larger detection window ( Figures 6 and 7).
• Greater breadth of data collected. Time-lapse data could also be analysed for cell migration and apoptosis as desired.

The limitations of this approach are the relatively low throughput of time-lapse proliferation analysis, which was done manually in this case, however computer assisted quantification might be possible. Another limitation specifically regarding use of zebrafish to study genes involved in proliferation is that not all human genes have zebrafish orthologues, which may restrict candidates for study in this model.

This study demonstrates the utility of time-lapse imaging zebrafish embryos to measure EC proliferation. Comparable *in vitro* models (HUVECs, HCAECs) allow real-time imaging of EC proliferation, but lack biological relevance. Whereas established *in vivo* models (mouse, pig) are biologically relevant yet cannot be used for real-time *in situ* monitoring of EC proliferation, and must be culled to isolate vascular tissue for analysis. By using the zebrafish embryo to study EC proliferation, researchers can achieve real-time *in situ* monitoring of EC proliferation in a biologically relevant model whilst replacing mammals and reducing or eliminating the use of terminal procedures, thus achieving partial replacement and refinement of use of animals in CVD research.

This time-lapse approach is more suitable than other methods for quantifying EC proliferation in zebrafish larvae and has applications for studying the mechanisms of proliferation and for screening to detect compounds that modify this process, we are currently using this approach to use zebrafish larvae to screen for genes involved in EC proliferation in response to blood flow. Whilst the manual counting approach used currently is low-throughput, future work could automate detection of proliferation using image macros to enable widespread use of this system for identifying genes involved in atherosclerosis

## Data availability

### Underlying data

OSF: Underlying data for ‘Quantifying endothelial cell proliferation in the zebrafish embryo’. https://www.doi.org/10.17605/OSF.IO/NV9XE.

The project contains the following underlying data:

Raw images of three embryos imaged using the PCNA method, three embryos imaged using the EdU method, and three embryos imaged using the timelapse method.

### Extended data

OSF: Extended data for ‘Quantifying endothelial cell proliferation in the zebrafish embryo’. https://www.doi.org/10.17605/OSF.IO/Y2SCW.

The project contains the following extended data:

Supplementary Figure 1: Dechorionation of zebrafish embryos. 1) Insert forceps into embryo chorion, be careful not to damage the embryo. 2) Gently open forceps to tear the chorion further. 3) Widen the forceps until the embryo has escaped the chorion.

Supplementary Figure 2: Preparing zebrafish embryos for microinjection. 1) Place a glass slide in a petri dish and tilt as shown. 2) Draw up embryos in a Pasteur pipette and decant onto the edge of the glass slide as shown. 3) Use the Pasteur pipette to draw up any excess liquid from the petri dish, leaving a row of zebrafish embryos against the glass slide.

## Reporting guidelines

OSF: ARRIVE 2.0 checklist for ‘Quantifying endothelial cell proliferation in the zebrafish embryo’. https://www.doi.org/10.17605/OSF.IO/Y2SCW.

Data are available under the terms of the Creative Commons Attribution 4.0 International license (CC-BY 4.0).

## References

[1] Weis SM Cheresh DA : Pathophysiological consequences of VEGF-induced vascular permeability. *Nature.* 2005;437:497–504. 10.1038/nature03987 16177780

[2] Kimmel CB Ballard WW Kimmel SR : Stages of embryonic development of the zebrafish. *Dev Dyn.* 1995;203:253–310, 10.1002/aja.1002030302 8589427

[3] Ekker SC : Morphants: a new systematic vertebrate functional genomics approach. *Yeast.* 2000;17:302–306. 10.1002/1097-0061(200012)17:4<302::AID-YEA53>3.0.CO;2-# 11119307 PMC2448384

[4] Ablain J Durand EM Yang S : A CRISPR/Cas9 vector system for tissue-specific gene disruption in zebrafish. *Dev Cell.* 2015;32:756–64. 10.1016/j.devcel.2015.01.032 25752963 PMC4379706

[5] Gilbert LA Larson MH Morsut L : CRISPR-mediated modular RNA-guided regulation of transcription in eukaryotes. *Cell.* 2013;154:442–51. 10.1016/j.cell.2013.06.044 23849981 PMC3770145

[6] Serbanovic-Canic J de Luca A Warboys C : Zebrafish Model for Functional Screening of Flow-Responsive Genes. *Arterioscler Thromb Vasc Biol.* 2017;37:130–143. 10.1161/ATVBAHA.116.308502 27834691 PMC5172514

[7] Weijts B Gutierrez E Saikin SK : Blood flow-induced Notch activation and endothelial migration enable vascular remodeling in zebrafish embryos. *Nat Commun.* 2018;9:5314. 10.1038/s41467-018-07732-7 30552331 PMC6294260

[8] Kugler EC van Lessen M Daetwyler S : Cerebrovascular endothelial cells form transient Notch-dependent cystic structures in zebrafish. *EMBO Rep.* 2019;20:e47047. 10.15252/embr.201847047 31379129 PMC6680135

[9] Lister JA Robertson CP Lepage T : nacre encodes a zebrafish microphthalmia-related protein that regulates neural-crest-derived pigment cell fate. *Development* .1999;126:3757–3767. 10433906 10.1242/dev.126.17.3757

[10] Savage AM Kurusamy S Chen Y : tmem33 is essential for VEGF-mediated endothelial calcium oscillations and angiogenesis. *Nat Commun* .2019;10:732. 10.1038/s41467-019-08590-7 30760708 PMC6374405

[11] Heckel E Boselli F Roth S : Oscillatory Flow Modulates Mechanosensitive klf2a Expression through trpv4 and trpp2 during Heart Valve Development. *Curr Biol.* 2015;25:1354–13561. 10.1016/j.cub.2015.03.038 25959969

[12] Roman BL Pham VN Lawson ND : Disruption of acvrl1 increases endothelial cell number in zebrafish cranial vessels. *Development.* 2002;129:3009–3019. 12050147 10.1242/dev.129.12.3009

[13] Leung AY Leung JC Chan LY : Proliferating cell nuclear antigen (PCNA) as a proliferative marker during embryonic and adult zebrafish hematopoiesis. *Histochem Cell Biol.* 2005;124:105–111. 10.1007/s00418-005-0003-2 16028068

[14] Luo N Li H Xiang B : Syndecan-4 modulates the proliferation of neural cells and the formation of CaP axons during zebrafish embryonic neurogenesis. *Sci Rep.* 2016;6:25300. 10.1038/srep25300 27143125 PMC4855150

[15] Salic A Mitchison TJ : A chemical method for fast and sensitive detection of DNA synthesis in vivo. *Proc Natl Acad Sci U S A.* 2008;105:2415–2420. 10.1073/pnas.0712168105 18272492 PMC2268151

[16] Xiong X Luo S Wu B : Comparative Developmental Toxicity and Stress Protein Responses of Dimethyl Sulfoxide to Rare Minnow and Zebrafish Embryos/Larvae. *Zebrafish.* 2017;14:60–68. 10.1089/zeb.2016.1287 27509300

[17] Li W Li H Sanders PN : The multifunctional Ca2+/calmodulin-dependent kinase II delta (CaMKIIdelta) controls neointima formation after carotid ligation and vascular smooth muscle cell proliferation through cell cycle regulation by p21. *J Biol Chem.* 2011;286:7990–7999. 10.1074/jbc.M110.163006 21193397 PMC3048686

[18] Zhang Y Song H Wu F Mu Q Jiang M Wang F Zhang W Li L Shao L Li S Yang L Zhang M Wu Q Tang D : Irisin Inhibits Atherosclerosis by Promoting Endothelial Proliferation Through microRNA126-5p. *J Am Heart Assoc.* 2016;5. 10.1161/JAHA.116.004031 27671318 PMC5079047

