# Supplementary material for: Quantifying endothelial cell proliferation in the zebrafish embryo
Source: F1000Res. 2021 Oct 11;10:1032. [Version 1] doi: 10.12688/f1000research.73130.1 (PMC9944168; doi:10.12688/f1000research.73130.1)
Supplement: Peer review history

# Peer review: Reviewer response for version 1

**DOI:** 10.5256/f1000research.76756.r156027

**Published:** 2023-02-21

**Authors:** Stephen J. White

**Affiliations:** 1 Department of Life Sciences, Manchester Metropolitan University, Manchester, UK

The Article by Bowley et al. describes a novel approach to quantify the relatively slow rates of endothelial proliferation in vivo, using zebrafish embryos, rather than in vitro models. The approach has several advantages over using other animal models to examine endothelial proliferation in vivo, including the potential use of zebrafish lines that use fluorescent reports for cell-type differentiation, the ability to examine proliferation pre-post heart beat, to determine any effects of flow and easy genetic manipulation through genome editing or knockdown with morpholinos. As such, this model provides a flexible platform to examine drug or gene regulation of (endothelial) proliferation.

 The text and methodology is clearly described and in sufficient detail to allow other scientists to replicate the experiments described here. As such it is a useful step forward to quantify in vivo proliferation while minimising the use of protected species. The platform is sufficiently flexible to be used to advance the field on this topic.

Is the rationale for developing the new method (or application) clearly explained?

Yes

Are the 3Rs implications of the work described accurately?

Yes

Is the description of the method technically sound?

Yes

If applicable, is the statistical analysis and its interpretation appropriate?

Yes

Are the conclusions about the method and its performance adequately supported by the findings presented in the article?

Yes

If any results are presented, are all the source data underlying the results available to ensure full reproducibility?

Yes

Are a suitable application and appropriate end-users identified?

Yes

Are sufficient details provided to allow replication of the method development and its use by others?

Yes

Reviewer Expertise:

Endothelial biomechanics and endothelial dysfunction

I confirm that I have read this submission and believe that I have an appropriate level of expertise to confirm that it is of an acceptable scientific standard.

# Peer review: Reviewer response for version 1

**DOI:** 10.5256/f1000research.76756.r96625

**Published:** 2022-11-01

**Authors:** Tzung Hsiai

**Affiliations:** 1 Division of Cardiology, Department of Medicine, David Geffen School of Medicine, University of California, Los Angeles, Los Angeles, CA, USA

In this method article, Bowley *et al.* determine the feasible method to quantify endothelial cell (EC) proliferation which regulates vascular formation and homeostasis in zebrafish embryos to investigate development via a comparison between three different comparative analyses: antibody, DNA labeling, and time-lapse imaging. Although rodent models are predominantly used to explore the mechanism of EC proliferation in cardiovascular disease or development, the authors pointed out there is a limitation - time-consuming, costly, and detrimental to animal welfare. The authors concluded that time-lapse imaging is accurate, precise, scalable, simple, and effective than the other two methods: antibody (PNCA) or DNA labeling (EdU) in EC proliferation study in the zebrafish embryo model. This method paper is necessary to approach EC proliferation in zebrafish and is well designed to compare three different methods, however, there are major and minor points that need to be improved.

1. The authors are focusing on the quantification of EC proliferation using three different methods, however, vascular endothelial growth factor (VEGF) is the most common marker for EC proliferation. Please address why the authors chose PCNA for detecting proliferating cell nuclear antigens and EdU for measuring DNA proliferation instead of VEGF, and it is necessary to be discussed.
2. EdU labeling is a well-established method to quantify S-phase cells. The authors performed EdU labeling in zebrafish embryos for 1 hour and showed that it did not label as many cells as they observed via tracking nuclear division over 6 hours (Figure 5). This does not translate to EdU labelling being unable to provide accurate measurements. Instead, it simply prompts a longer period of EdU incubation. The authors argue that the high concentration of DMSO used in the labeling solution is toxic to embryos for long-term incubation, however, there is no necessity of using DMSO during EdU incubation as EdU is soluble in aqueous buffers. The authors should perform extra experiments on this.
3. On the other hand, there are large amounts of EdU+ cells (green) colocalization with ECs (red) in Figure 3. The authors should also test their hypothesis at different regions of the vascular network, not just ISV.
4. In Figure 2, to see the clear effects of tnnt2 morphant (inhibition of heart contraction) in EC proliferation, the control group (control morphant) should be presented. The authors also need to provide negative control images with only secondary antibodies to validate the staining, as virtually every cell in the image was red, meaning PCNA+. However, PCNA staining should give fewer labeled cells than EdU staining and time-lapse tracking, according to the authors. It can also be that the PCNA antibody is not working well. The authors need to address this.
5. In addition, to rely on the comparison, Figures 2 and 3 should be offered the same enlargement.
6. In Figure 5, two-way ANOVA is an advisable statistical method among the groups.
7. The graphical abstract (Figure 7) is arduous to comprehend because of a lack of a rationale for the limitation of immunostaining and EdU labeling. The authors should offer the reference or evidence to elaborate the experimental design.

Is the rationale for developing the new method (or application) clearly explained?

Yes

Are the 3Rs implications of the work described accurately?

Yes

Is the description of the method technically sound?

Yes

If applicable, is the statistical analysis and its interpretation appropriate?

Partly

Are the conclusions about the method and its performance adequately supported by the findings presented in the article?

Yes

If any results are presented, are all the source data underlying the results available to ensure full reproducibility?

Partly

Are a suitable application and appropriate end-users identified?

Yes

Are sufficient details provided to allow replication of the method development and its use by others?

Yes

Reviewer Expertise:

NA

I confirm that I have read this submission and believe that I have an appropriate level of expertise to confirm that it is of an acceptable scientific standard, however I have significant reservations, as outlined above.

# Peer review: Reviewer response for version 1

**DOI:** 10.5256/f1000research.76756.r118456

**Published:** 2022-02-07

**Authors:** Christopher George

**Affiliations:** 1 Institute of Life Sciences, Swansea University, Swansea, UK

The authors’ clearly set out the context for their study; developing a non-invasive experimental system to accurately capture spatial and temporal information on EC proliferation in the vasculature would be important step forward. Three techniques are explored for achieving these aims. Two (PCNA immunostaining, EdU incorporation) do not appear to work for the intended purpose but it is not immediately clear from the data shown why. More ‘negative’ data would be very helpful in setting out the limitations of these techniques so that others can appreciate why these approaches ‘failed’. One technique, time-lapse imaging of nuclear division (where labelling driven by a EC-specific promoter) does appear to have some future applicability, although the technique, at present, is limited to manual counting and and very low numbers of experiments are reported in support of this approach.

 There are a number of issues with the current version of the manuscript that require attention:

**Abstract**

 The abstract refers specifically to n=24 experimental units, where in the example given, n=4 comprise controls and then four groups of n=5 ‘treated’ groups. These numbers seem rather arbitrary and are not referred to in the paper.

**Introduction / General**

 It would be helpful to include more detail on how the ZF embryo CV system recapitulates key features of mammalian circulatory system. Please provide clarity (and perhaps illustrative detail) on how ZFe’s “complete cardiovascular system” has a “simplicity comparable with that of two dimensional models in vitro” and “comparable relevance to mammalian in vivo models”. This might help uptake by those researchers typically using mammalian models of vascular plasticity.

 Blebbing of nuclei is not a unique marker of apoptosis. Autophagy and other activation of modes of cellular degradation are characterised by blebbing. The authors imply that apoptosis is the sole mechanism of cell death in ZF embryos. Is this correct? No data on cell death is reported in the manuscript so the references to apoptosis don’t seem to have much relevance in the context of the paper.

**Methods**

 More methodological detail / explanation should be included:

- How are male and female zebrafish distinguished?
- Does the Nacre line have identical vascularization as wild-type?
- You state that “both transgenic lines use Nacre as background” but the study uses three TG lines.
- How leaky, or not, is the EC-specific promoter. Can you be sure that expression is only in EC lineages? This is a key issue since your method relies on typing ‘EC-specific’ expression.
- A reference to the inhibition of contractility by tnnt2a morpholino should be given.
- It is presumed that that TG-EGFP was used for PCNA and TG-mCherry was used for EdU to avoid the spectral overlap of fluorophores in each instance? This should be stated.
- More details on how the commercial EdU labelling kit results in the generation of a detectable 488nm signal should be included. What is the concentration of saponin? What is the range of EdU signal developed (i.e. time-dependent accumulation)? How homogeneous is this across cells/FACS populations?
- Brightfield microscopy is included as the means for discriminating dead or undeveloped embryos are identified. How? No brightfield imaging is included in the paper.
- Please define Light Sheet microscopy as LSM (to avoid confusion with the common use of LSM as (confocal) laser scanning microscopy.
- The statement that “587nm excitation is active”. Please include the excitation source and what is meant by “active”.
- The methods include non-terminal anaesthesia using E3 medium (“Fluorescent microscopy”) or E3 medium and tricaine (“Light-sheet microscopy”). Please clarify. What is the final concentration of tricaine used in anaesthesia?
- Statistics. Please check that two-way ANOVA is an appropriate statistical test for data where n=3 (i.e. is distribution normal?).
- Microinjection - it is appreciated that the settings are specific for the Sutter puller but it would be helpful to include units as applicable (e.g. heat, 475 *degrees C*?).
- Into which ZF embryo region is the morpholino injected?

**Results**

- Figure 1. Perhaps this cartoon could be shown alongside actual staining of these sections in a representative embryo.
- Figure 2. According to the Methods section, confocal imaging fields were approximately 869 x 869 microns. Figure 2 is approx. 250 x 250 microns. It would be helpful to show the entire imaging field and then the ‘zoom’ into the region shown in Figure 2. In my copy for review, Figure 2 has thin yellow lines which are not explained and the yellow arrows do not appear to correspond to co-stained cells. Figure 2 does not support the use of PCNA as being a good marker for EC proliferation (indeed you state this, indirectly, in the Discussion) but its not immediately clear what it *does *show. It would be helpful too if regions, like in the cartoon in Figure 1 were included on Figure 2.
- Figure 3 should be presented at the same magnification/zoom as Figure 2. Figure 3 should also be better integrated with your conclusion that EdU labelling was ruled out of contention as a technique for quantifying EC proliferation. Do you have any images showing (visually) the effect of 15% DMSO on sample morphology beyond 1 hour? It is also somewhat misleading to state, as you do in Figure 7, that EdU labelling is limited by low detection of EC proliferation. The problem seems to be that the reagent is toxic which prevents longer-term imaging. It needs to made more clear that EdU is of limited use for mapping cellular proliferation occurring on a time-scale incompatible with reagent toxicity.
- Figure 4. Your findings suggest that to capture sufficient EC proliferation, an experimental window of up to 12 hours is required. Figure 4 only shows a plus-15min window. Some idea of how time-lapse capture of EC proliferation changes with time would be very helpful. Also, the arrowheads appear squashed and point away from the area you mean to depict.
- Figure 5 shows the number of proliferation events but this is not that useful. It would be much better if you showed this the number of as the proportion or percentage of mCherry cells scrutinized. ‘n’ is very low here and statistical analysis is not convincing (although the effect is obvious).

**Discussion**

- It is not possible to conclude, from the data shown, that nuclei-imaging is “accurate” (Figure 7). This will need much more data and deeper modes of analysis.
- As you note, “computer assisted quantification” would augment your approach, but this phrase is vague. What would you want to focus on, and what would need to be taken into account / eliminated etc. More discussion on how you see this approach being potentially worked-up into a medium- to high-throughput screening platform would be helpful.

**Other**

- Zebrafish welfare and use section. The second-to-last sentence in the first paragraph needs splitting into two sentences. It would be helpful to define what constitutes an “adverse event” here.
- PCNA and EDU should be described in full at first mention. Has PCNA ever been used as a marker of proliferation during zebrafish CV development? (You state kidney but what about CV…….)
- The phrase “humanely destroyed using bleach” is inappropriate. It might be better worded as “embryos were destroyed using bleach”. Ditto “euthanised” on pages 5 and 8.
- The Supplemental Figures do not add to the article and should be omitted. Videos of the actual procedures would be much better.

Is the rationale for developing the new method (or application) clearly explained?

Yes

Are the 3Rs implications of the work described accurately?

Yes

Is the description of the method technically sound?

Partly

If applicable, is the statistical analysis and its interpretation appropriate?

No

Are the conclusions about the method and its performance adequately supported by the findings presented in the article?

Partly

If any results are presented, are all the source data underlying the results available to ensure full reproducibility?

Partly

Are a suitable application and appropriate end-users identified?

Yes

Are sufficient details provided to allow replication of the method development and its use by others?

Partly

Reviewer Expertise:

Network behaviour of cardiovascular cell types / cell imaging / imaging technologies / signal decoding. Referee suggested by the NC3Rs for their scientific expertise and experience in assessing 3Rs impact.

I confirm that I have read this submission and believe that I have an appropriate level of expertise to confirm that it is of an acceptable scientific standard, however I have significant reservations, as outlined above.
